# In Vitro Evaluation of Sunscreen Safety: Effects of the Vehicle and Repeated Applications on Skin Permeation from Topical Formulations

**DOI:** 10.3390/pharmaceutics10010027

**Published:** 2018-02-27

**Authors:** Lucia Montenegro, Rita Turnaturi, Carmela Parenti, Lorella Pasquinucci

**Affiliations:** 1 Department of Drug Sciences, Pharmaceutical Technology Section, University of Catania, Viale A. Doria 6, 95125 Catania, Italy; 2 Rita Turnaturi Department of Drug Sciences, Medicinal Chemistry Section, University of Catania, Viale A. Doria 6, 95125 Catania, Italy; rita.turnaturi@tiscali.it; 3 Carmela Parenti Department of Drug Sciences, Pharmacology and Toxicology Section, University of Catania, Viale A. Doria 6, 95125 Catania, Italy; cparenti@unict.it; 4 Lorella Pasquinucci Department of Drug Sciences, Medicinal Chemistry Section, University of Catania, Viale A. Doria 6, 95125 Catania, Italy; lpasquin@unict.it

**Keywords:** sunscreen, topical formulation, safety, skin permeation, octyl methoxycinnamate, butylmethoxydibenzoylmethane, repeated application

## Abstract

The evaluation of UV-filter in vitro percutaneous absorption allows the estimation of the systemic exposure dose (SED) and the margin of safety (MoS) of sunscreen products. As both the vehicle and pattern of application may affect sunscreen safety and efficacy, we evaluated in vitro release and skin permeation of two widely used UV-filters, octylmethoxycinnamate (OMC) and butylmethoxydibenzoylmethane (BMBM) from topical formulations with different features (oil in water (O/W) emulsions with different viscosity, water in oil (W/O) emulsion, oils with different lipophilicity). To mimic in-use conditions, we carried out experiments repeating sunscreen application on the skin surface for three consecutive days. BMBM release from all these vehicles was very low, thus leading to poor skin permeation. The vehicle composition significantly affected OMC release and skin permeation, and slight increases of OMC permeation were observed after repeated applications. From skin permeation data, SED and MoS values of BMBM and OMC were calculated for all the investigated formulations after a single application and repeated applications. While MoS values of BMBM were always well beyond the accepted safety limit, the safety of sunscreen formulations containing OMC may depend on the vehicle composition and the application pattern.

## 1. Introduction

The increasing awareness that unprotected exposure to ultraviolet (UV) radiation is a major causal factor in the development of skin cancer has led to a rise in the use of sunscreen products [1,2]. The knowledge of the potential for human systemic exposure is a key factor in the safety assessment of UV-filters used in marketed products. Theoretically, sunscreen agents’ activity should be restricted to the skin surface or within the upper layers of the stratum corneum, to avoid adverse systemic effects due to their penetration into the underlying viable tissues. However, many studies regarding the skin penetration and permeation of topically applied UV-filters pointed to the ability of some sunscreen agents to overcome the skin barrier, thus arriving at the systemic circulation [3]. In vivo and in vitro percutaneous absorption studies on benzophenone-3 (BP-3), a common sunscreen agent, showed that this compound was absorbed through the skin both in human volunteers and in animal models [4,5]. In vivo experiments on piglets showed that oxybenzone permeated through the skin and was detected in plasma for up to 48 h after one single topical application [6]. Janjua et al. [7] reported an investigation on three sunscreens, BP-3, octyl-methoxycinnamate (OMC), and 3-(4-methylbenzylidene) camphor (4-MBC) after topical application as cream formulation in healthy volunteers. These UV-filters were absorbed through the skin and were recovered in urine. These findings, together with some reports on the estrogenic activity of certain UV-filters after their topical application [8,9,10], have given rise to a great concern about the safety of using sunscreen products. Therefore, several researchers [11,12] calculated the margin of safety (MoS) of UV-filters by comparing the potential human systemic exposure with the no adverse effect level (NOAEL) from in vivo toxicity studies. The MOS expresses the ratio between the NOAEL for the critical effect and the systemic exposure dosage (SED), which can be evaluated theoretically or experimentally. MoS value should be equal to or greater than 100 to declare that a substance is safe for use. Therefore, the accepted safety limit is a MoS value equal to or greater than 100. The results of these in vivo toxicity studies highlighted that the potential human risk from sunscreen use is negligible, as their actual safety margins are considerably higher than calculated values. In vitro studies on the penetration of five commonly used sunscreen agents (avobenzone, octinoxate, octocrylene, oxybenzone and padimate O) through human skin from mineral oil supported this consideration by pointing out that the human viable epidermal levels of sunscreens were too low to cause any significant toxicity to the underlying epidermal cells [13]. As sunscreen efficacy may depend on vehicle formulation, some researchers investigated the vehicle effects on UV-filters skin penetration and permeation [14,15,16,17,18,19]. In a previous study, we evaluated the safety of six commercial sunscreen emulsions, with medium-high sun protection factor (SPF), containing two of the most widely used UV-filters, butyl methoxydibenzoylmethane (BMBM) and OMC, by evaluating their in vitro skin permeation and their MoS values [20]. The results of this study showed that MoS values of these products were considerably beyond the limit accepted for safe products. Recently, Hojerová et al. [21] reported that skin shaving and sunscreen reapplication could affect in vitro skin permeation of benzophenone-3 and ethylhexyl triazone from a silicone-based water-in-oil emulsion.

Although both the vehicle and the pattern of application may significantly affect drug percutaneous absorption [22,23,24,25], there are only a small amount of data about the vehicle effects on the percutaneous absorption of UV-filters under in use conditions, in particular after repeated applications for consecutive days. Therefore, in this work, we assessed the in vitro skin permeation of two of the most commonly used UV-filters, OMC (UVB-filter) and BMBM (UVA-filter) from different topical vehicles, oil in water (O/W) cream, water in oil (W/O) cream and oily lotions with different lipophilicity, after repeated applications for three consecutive days. Furthermore, from in vitro results, we calculated MoS values for all these formulations to estimate their safety after in vivo topical application.

The results of in vitro skin permeation studies showed that MoS values of BMBM were above the accepted safety limit for all the investigated vehicles while the safety of formulations containing OMC may be affected by their composition.

## 2. Materials and Methods

### 2.1. Materials

Cetearyl isononanoate (Cetiol SN^®^), decyl oleate (Cetiol V^®^), caprylic/capric triglyceride (Myritol 318^®^), glyceryl stearate (and) ceteareth-20 (and) ceteareth-12 (and) cetearyl alcohol (and) cetyl palmitate (Emulgade SE-PF^®^), sorbitan stearate (Dehymuls SMS^®^), octylmethoxycinnamate (Uvinul MC 80^®^, OMC) and butylmethoxydibenzoylmethane (Uvinul BMBM^®^, BMBM) were a kind gift from BASF Care Creations (Monheim, Germany). Beeswax, glyceryl monostearate (GMS), jojoba oil, almond oil and butylhydroxytoluene (BHT) were purchased from Esperis (Milan, Italy). Mineral oil, isopropyl myristate (IPM), glycerin, squalane and disodium EDTA were supplied by Galeno (Prato, Italy). Methylchloroisothiazolinone and methylisothiazolinone (Kathon CG^®^), and imidazolidinyl urea (Gram 1^®^) were kindly supplied by Sinerga (Gorla Maggiore, Italy).

Cellulose acetate membranes (Spectra/Por CE; Mol. Wt. cut off 3.000) were bought from Spectrum (Los Angeles, CA, USA). Acetonitrile and water used in the HPLC procedures were of LC grade and were obtained from Merck (Milan, Italy). All other reagents were of analytical grade.

### 2.2. Preparation of Emulsions 1–3

The composition of emulsions 1, 2 (O/W) and 3 (W/O) is reported in Table 1 and Table 2, respectively. All the oil (phase A) and aqueous phase ingredients (phase B) were placed in separate glass containers and heated to 70 °C. The water phase was added to the oil phase under vigorous stirring (Turbomixer Silverson SL2, Silverson Machines Inc., East Longmeadow, MA, USA). The resulting emulsion was then cooled to 40 °C and the preservatives (phase C) were added. The formulation was then cooled to room temperature under slow and continuous stirring. The samples were stored in airtight glass jars at room temperature sheltered from the light until used. No significant alteration of the organoleptic properties of these formulations was observed during their storage at room temperature and in the dark for three months.

Viscosity of emulsions 1–3 was determined 48 h after their preparation. A Mettler Rheomat RM 260 viscosimeter (Mettler_Toledo, Milan, Italy) was used to measure the viscosities (mPas) of the emulsions prepared using a MSDIN 125 spindle at shear rate 3 for 10 s. Samples of the emulsions were left to settle over 30 min at room temperature before taking the measurements.

### 2.3. Preparation of Oil Vehicles 4–6

Oil vehicle 4–6 composition and oil required hydrophilic lipohilic balance (HLB_r_) values are shown in Table 3. Oil vehicles 4–6 were prepared by mixing the weighted amount required of each oil at room temperature. Due to their different composition, the oil vehicles showed the following HLB_r_ values: oil 4 = 4.38, oil 5 = 5.29, oil 6 = 6.59. The samples were stored in airtight glass jars at room temperature sheltered from the light until used. No significant alteration of the organoleptic properties of these formulations was observed during their storage at room temperature and in the dark for three months.

### 2.4. In Vitro Release Experiments

Sunscreen release rates from the cosmetic vehicles under investigation were measured through cellulose membranes by means of Franz-type diffusion cells (Laboratory Glass Apparatus, Berkeley, CA, USA). This technique is regarded as a suitable method to assess drug release from topical formulations [26].

The cellulose membranes were moistened by immersion in distilled water for 1 h at room temperature before being mounted in Franz-type diffusion cells whose surface area was 0.75 cm^2^ and whose receiving chamber volume was 4.5 mL. The receiving solution consisted of water/ethanol (50/50 *v*/*v*) for ensuring pseudo-sink conditions by increasing sunscreen solubility in the receiving phase [19]. The receptor phase was constantly stirred and thermostated at 35 °C to maintain the membrane surface at 32 °C. Each formulation (20 mg/cm^2^) was applied on the membrane surface and the experiments were run for 24 h. At intervals (0, 2, 4, 6, 8, 24 h), samples of the receiving solution (200 μL) were withdrawn and replaced with an equal volume of receiving solution pre-thermostated at 35 °C. Samples of the receptor phase were analyzed by the HPLC method described below to determine their sunscreen content. Each experiment was performed in triplicate.

### 2.5. In Vitro Skin Permeation Experiments

In vitro permeation experiments through human skin were performed as reported in the Organisation for Economic Co-operation and Development (OECD) Guidelines 156, with minor changes [27]. Stratum corneum and epidermis (SCE) membranes were prepared as described by Kligman and Christophers [28]. SCE membranes were used to evaluate sunscreen skin permeation since the dermis in vitro can act as an artificial barrier to the penetration of lipophilic compounds [29]. Furthermore, this kind of membrane is regarded a suitable model to assess in vitro skin permeation of UV-filters [30]. Briefly, subcutaneous fat was carefully trimmed from samples of adult human skin (obtained from abdominal plastic surgery; mean age 35 ± 6 years) and the skin was immersed in distilled water at 60 ± 1 °C for 2 min, after which stratum corneum and epidermis (SCE) were removed from the dermis using a blunt scalpel blade. This technique allowed a neat separation of the SCE from the dermis at the dermal–epidermal junction, and no residual dermis could be found in these SCE membranes. SCE membranes were dried in a desiccator at approximately 25% RH and stored at 4 °C until used, as described by Swarbrick et al. [31]. Samples of dried SCE were rehydrated by immersion in distilled water for 1 h prior to the experiment. SCE samples were mounted in the same Franz-type diffusion cells described above. The receiving compartment was filled with water/ethanol (50/50 *v*/*v*) which was constantly stirred and thermostated at 35 °C to maintain the membrane surface at 32 °C. The use of such a receiving solution has already been described in the literature to ensure solubility of poor water-soluble compounds in in vitro percutaneous absorption studies [32]. As requested by the OECD guidelines [27], we checked that no alteration of skin permeability occurred throughout the experiments due to the receptor fluid we used. Therefore, preliminary experiments were performed to assess SCE samples for barrier integrity by measuring in vitro permeability coefficient of [^3^H] water in the same experimental conditions used for in vitro permeation experiments on topical vehicles [20]. As we performed these experiments using a single application protocol and a repeated application protocol, in vitro permeability coefficient of [^3^H] water was determined using both protocols. For evaluating skin permeation after a single application, [^3^H] water (500 μL) was applied on the skin surface. Samples of the receiving solution were withdrawn after 0, 2, 4, 6, 8, and 24 h and replaced with an equal volume of receiving solution pre-thermostated at 35 °C. For repeated application experiments, on day 1, [^3^H] water (500 μL) was applied on the skin surface for 8 h. Then, the remaining water on the skin surface was gently wiped off with a cotton swab until the skin surface was dry and the skin was left untreated for the following 16 h. Samples of the receiving solution were withdrawn after 0, 2, 4, 6, 8, and 24 h and replaced with an equal volume of receiving solution pre-thermostated at 35 °C. On day 2 and 3, [^3^H] water was applied again on the skin surface and the same procedure described for day 1 was followed. On day 4 of the repeated application protocol, we observed a slight but significant increase of skin permeability. Therefore, we ended this protocol on day 3. Tritiated water skin permeation was quantified as previously reported [20]. The value of permeability coefficient for tritiated water for both protocols was 1.7 ± 0.3 × 10^−3^, which was in good agreement with that reported for samples with normal skin permeability [33,34]. In the single application protocol, each formulation (20 mg/cm^2^) was applied to the skin surface and the experiments were run for 24 h. At intervals (0, 2, 4, 6, 8 and 24 h), 200 μL of the receptor phase were withdrawn and replaced with an equal volume of receiving solution pre-thermostated at 35 °C. In the repeated application protocol, experiments were performed using the following procedure: on day 1, 20 mg/cm^2^ of each formulation were applied on the skin surface. After 8 h, the residual formulation on the skin surface was gently wiped off with a cotton swab until the skin surface was clean and the skin was left untreated for the following 16 h. Samples of the receiving solution were withdrawn at 0, 2, 4, 6, 8, 22 and 24 h and replaced with an equal volume of receiving solution pre-thermostated at 35 °C. On day 2, immediately after the withdrawal of the 24 h sample from the receiving solution, 20 mg/cm^2^ of each formulation were applied again on the skin surface and the same procedure described on day 1 was followed. The repeated application protocol lasted 3 days. Due to sunscreen photoinstability, all experiments were performed avoiding light exposure.

Samples of the receiving solution were analyzed by the HPLC method described below to determine their UV-filter content. Each formulation was tested in triplicate on three different skin specimens.

### 2.6. High Performance Liquid Chromatography (HPLC) Analyses

The HPLC system consisted of a Varian ProStar model 230 (Varian, Milan, Italy) with an auto-sampler Varian model 410 and a Galaxie 1.9 software for data elaboration. All the chromatographic analyses were performed using a Waters Simmetry, 4.6 × 25 cm reverse phase column (C_18_) (Waters, Sesto San Giovanni, Italy) and a mobile phase consisting of acetonitrile/water (80:20 *v*/*v*) under isocratic conditions at room temperature. The flow rate was 1.0 mL/min. 20 μL of each sample was injected and the column effluent was monitored continuously to detect OMC (310 nm) and BMBM (360 nm). The amount of UV-filter contained in each sample was calculated by reporting the peak area of the sample on a standard calibration curve that was built up by relating known concentrations of UV-filters with the respective peak areas. No interference of the other formulation components was observed. The sensitivity of the method was 0.1 μg/mL for both UV-filters.

### 2.7. Data Analysis

UV-filter flux (μg/cm^2^/h) through the cellulose membrane or through the skin was calculated by plotting the cumulative amount of compound released or permeated against time and dividing the slope of the steady-state portion of the graphs by the area through which diffusion took place. The lag time was determined from the x-intercept values of the regression lines.

Systemic exposure dosage (SED) was estimated as reported by Søeborg et al. and by the Scientific Committee on Consumer Safety (SCCS) 2016 [35,36] using the following equation:

(1) $$\mathrm{SED}\left(\mathrm{mg}/\mathrm{Kg}\;\mathrm{body}\;\mathrm{weight}/\mathrm{day}\right)=\frac{{\mathrm{DA}}_{a}\left(\mu g/{\mathrm{cm}}^{2}\right)\times 10^{-3}\;\mathrm{mg}/\mu g\times \mathrm{SSA}\left({\mathrm{cm}}^{2}\right)\times F\left({\mathrm{day}}^{-1}\right)}{60\;\mathrm{Kg}}$$

where DA_a_ (μg/cm^2^) is the dermal absorption reported as amount/cm^2^, resulting from in vitro skin permeation experiments, SSA is the skin surface area expected to be treated with the formulation under investigation, F is the frequency of application of the investigated formulation and 60 Kg is the default human body weight.

According to Søeborg et al. [35], an area of 15,000 cm^2^ corresponding to treatment of 83% of the skin was used to simulate the worst scenario of the application of a body formulation. A single application of cream per day (F = 1) was used for all calculations.

To estimate the margin of safety (MoS), the SED was compared to the NOAEL:

MoS = NOAEL/SED(2)

where NOAEL is the no observed adverse effect level determined in animal toxicity studies. NOAEL for OMC and BMBM was obtained from the literature [12] and its value, determined in rats, was 450 mg/Kg/day for both active compounds.

Results were expressed as mean values ± standard deviation (S.D.) and Student’s *t*-test was used to evaluate the significance of the difference between mean values. Values of *p* < 0.05 were considered statistically significant.

## 3. Results and Discussion

### 3.1. In Vitro Release Experiments

The safety and efficacy of UV-filters depend on their ability to remain on the skin surface after topical application of a sunscreen product, without (or with minimum) permeating into the deeper skin layers. The vehicle formulation may play a significant role in determining sunscreen safety and efficacy as some formulation ingredients can influence both the technological and physico-chemical properties of the product such as UV-filter absorbing capacity (wavelength of absorption and molar extinction coefficient), spreadability, thickness of the film formed on the skin, SPF, water resistance and active ingredient skin penetration/permeation [37]. In particular, emollients, solvents, film formers and occlusive substances could increase skin hydration, thus favoring skin penetration/permeation of formulation ingredients [38]. Therefore, to address risk assessment issues, in vitro and in vivo percutaneous absorption studies under conditions mimicking the actual exposure are highly recommended.

To perform in vitro percutaneous absorption studies, OECD guidelines (OECD, 2011) recommended the use of finite dosing, as this experimental protocol more closely resembles “in use” situations. In particular, as far as UV-filters are concerned, Cosmetics Europe—The Personal Care Association (formerly COLIPA) prescribes an application dose of 2 mg/cm^2^. However, this application dose has not been indicated to replicate the consumer use but to guarantee the inter- and intra-laboratory reproducibility of the SPF test [39].

As the manufacturers suggest that the consumers apply repeatedly sunscreen products to maintain a constant amount of UV-filter on the skin surface, we thought that the use of larger doses would more closely resemble the correct application regimen of sunscreen formulations indicated by the producers.

Therefore, in this study, we used the infinite dose technique to evaluate OMC and BMBM in vitro release and skin permeation as the application of larger doses would maintain a constant amount of UV-filter on the skin surface, preventing the depletion of the active ingredient that is typical of the finite dose technique. In previous works [18,20,40], we have already used the infinite dose technique to assess in vitro skin permeation of UV-filters from different cosmetic emulsions as this technique allowed maintaining a constant driving force for the permeation process during the experiment. Furthermore, Walters et al., 1997 [41], studying in vitro skin permeation of the UV-filter octyl salycilate from topical vehicles, did not observe a significant difference between the data obtained using a finite and an infinite dose technique.

As reported in literature [42], the first step in the percutaneous absorption process of an active ingredient is its release from the vehicle. Therefore, prior to performing skin permeation studies, we carried out in vitro release experiments on each vehicle under investigation. In vitro release data obtained from vehicles 1–6 are reported in Table 4.

With regard to BMBM, similar amounts were released after 24 h from the vehicles 1, 2, 3, 5 while no release was observed from the vehicles 4 and 6. As we could not detect BMBM in the receptor phase up to 8 h, we could not calculate the release rate of this UV-filter from all the vehicles under investigation. It is interesting to note that all emulsions (vehicles 1, 2, 3) provided similar BMBM release, regardless of their different features. Vehicles 1 and 2 were O/W emulsions with viscosity values of 28.000 and 8.200 mPas, respectively, while vehicle 3 was a W/O emulsion with a viscosity value of 30.100 mPas. On the contrary, among the oily vehicles (4, 5, 6), only formulation 5 was able to release a detectable amount of BMBM. As reported in Section 2.3, these oily vehicles showed different lipophilicity due to their diverse composition. In particular, BMBM release was observed only from the vehicle with intermediate lipophilicity, thus suggesting that BMBM release could be modulated by choosing suitable ingredients to formulate oily topical products.

As shown in Table 4, very low percentages of BMBM were released after 24 h from vehicles 1, 2, 3 and 5. Similar low percentages of the applied dose released have already been observed for BMBM in previous studies on various types of emulsions and oily vehicles [20].

**Figure 1:**
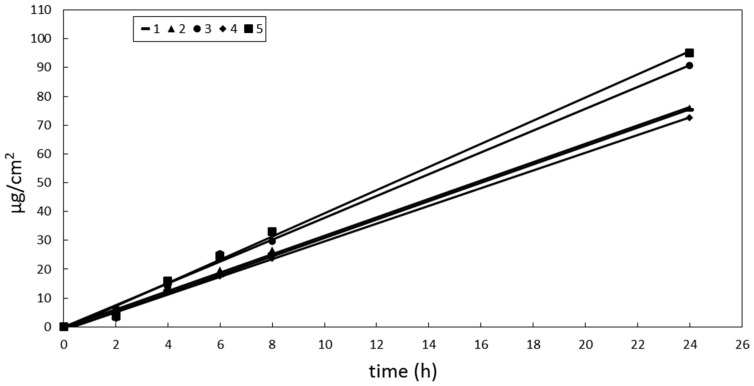
Cumulative amount of OMC released from vehicles 1–5 *vs* time. Standard error bars were omitted for clarity. The maximum coefficient of variation (CV = SD/mean × 100%) of the data was 18.

OMC showed a different pattern of release in comparison to BMBM. The cumulative amount of OMC released after 24 h from the vehicles under investigation decreased in the following order: 5 > 3 > 2 ≅ 1 ≅ 4 > 6. However, the amount of OMC released from the vehicle 6 was too small to be detected in the receiving solution up to 8 h, thus preventing us from calculating OMC release rate from this formulation. Plotting the cumulative amount of OMC released from vehicles 1–5 *vs* time, a linear relationship was obtained for all formulations (Figure 1). Emulsions 1 and 2 (O/W emulsions) showed similar OMC release rates that were lower than that observed from emulsion 3 (W/O). These differences in OMC release rates could be attributed to the presence of a lipophilic barrier to active compound release from oil droplets of O/W systems, which could slow the delivery of a lipophilic molecule distributed in the internal oily phase. Other authors have already put this hypothesis forward [43]. However, these different release rates did not affect the lag time as all formulations provided similar values (lower that 1 h).

As shown in Table 4, OMC release pattern from oily vehicles was very different from that obtained for BMBM. With OMC being more lipophilic that BMBM (see Table 5), a lower OMC release from the highest lipophilic vehicle could be expected. On the contrary, OMC showed a similar release from oily vehicles 4 and 5 and emulsions 1–3. The results of in vitro release studies showed that the type of external phase and the viscosity of the vehicle did not seem to play a significant role in determining OMC and BMBM release. Therefore, other factors, such as interactions among UV-filters and formulation ingredients, could be involved in determining OMC and BMBM release from these formulations, apart from the lipophilicity of the vehicle and active ingredient.

### 3.2. In Vitro Skin Permeation Experiments

As the physico-chemical properties are fundamental to determine UV-filter ability to penetrate through the skin, Watkinson et al. attempted to develop a suitable model to predict their percutaneous absorption [44]. Recently, Ates et al. proposed a novel approach to identify active ingredients with low skin permeation based on their physicochemical properties and their in vitro skin permeation [45]. In particular, if at least two of the following criteria apply—(a) molecular weight < 180 Da; (b) Log Pow > 0.3; (c) melting point < 100 °C; (d) topological polar surface area < 40 Å^2^—the molecule should show high skin permeation. According to this model, both BMBM and OMC, due to their physicochemical properties (see Table 5), should be able to permeate efficiently through the skin.

On the contrary, our skin permeation data revealed that BMBM was a poor skin permeant both after single application (Table 6) and after repeated applications (Table 7) in the vehicles under investigation.

As expected, no skin permeation of BMBM was observed from the formulations that did not provide any release of this UV-filter. From the analysis of the data reported in Table 6 and Table 7, it was evident that the rate-limiting step in BMBM percutaneous absorption process was its release from the vehicle. Therefore, these results highlighted the key role of the vehicle in determining the ability of an active ingredient to permeate through the skin. Furthermore, it is interesting to note that a slight increase of the cumulative amount of BMBM permeated through the skin on the second day of the repeated application protocol occurred without any further increase on the third day. Unfortunately, we could not extend the experiments because of permeability alterations of the skin samples after three days of exposure to the receiving medium. The increase of BMBM skin permeation could be likely due to the occlusive properties of the vehicles 3–6 or to the presence of IPM, a well-established skin penetration enhancer [47], in non-occlusive vehicles such as emulsions 1 and 2.

The data illustrated in Table 6 and Table 7 revealed a significant influence of the vehicle on OMC skin permeation. Unlike BMBM, the amount of OMC permeated in the single application protocol allowed us to calculate its flux through the skin and the lag time values for all formulations, apart from vehicle 6. From this last vehicle, no OMC permeation occurred (after single application and after repeated applications), likely due to its low release (see Table 4). OMC skin permeation (expressed as cumulative amount permeated after 24 h and as flux through the skin) from the vehicles 1–5 decreased in the following order: 5 > 3 > 2 ≅ 1 ≅ 4. Considerations similar to those reported for in vitro release experiments about the effects of vehicle viscosity and hydrophilicity apply to in vitro skin permeation studies.

By analogy with BMBM, the repeated applications protocol provided an increase of OMC skin permeation after two days with no further increase after three days.

In the repeated application protocol, we determined the cumulative amount of UV-filter permeated immediately after the removal of the formulation from the skin surface (i.e., 8 h after its application) and 16 h after its removal. The cumulative amount of OMC permeated 16 h after the removal of the vehicle was higher than that determined 8h after its application because, although the vehicle was removed, the amount of UV-filter already penetrated into the skin layers was released in the receiving medium. To evaluate if all OMC was released from the skin, we withdrew samples of the receiving solution 14 h after removing the vehicle, finding no significant difference in comparison with the UV-filter content in samples withdrawn after 16h. Therefore, we calculated indirectly the content of each UV-filter in the SCE membranes. As shown in Table 8, the amount of OMC in the SCE reflected that permeated through the skin. We could not calculate the content of BMBM in the SCE membranes using this method because the cumulative amount of BMBM permeated immediately after the removal of the formulation from the skin surface (i.e., 8 h after its application) was not significantly different from that permeated 16 h after the removal of the vehicle.

From skin permeation data, we calculated SED and MoS values for OMC and BMBM, as reported in Section 2.7 (see Table 9).

For formulations that did not provide any UV-filter skin permeation, MoS values could not be calculated but they could be regarded as tending to infinity because SED value was equal to zero.

BMBM MoS values were considerably higher than the accepted safety limit (>100) from all formulations under investigation after single application or repeated applications. On the contrary, the vehicles 1, 2, 3, and 5 provided OMC MoS values under the accepted safety limit after single application but, apart from vehicle 5, they could be regarded as safe after repeated applications. Furthermore, we could infer that applying an amount of formulation 10-folds lower than that used in this study, MoS values could be well beyond the limit accepted for safe products.

## 4. Conclusions

The results of this study support the safety of conventional topical vehicles containing OMC and BMBM as sunscreen agents. Recently, lipid-based nanoparticles have been proposed as carriers for topical active ingredients including UV-filters [48,49,50]. Therefore, further studies have been planned to evaluate the safety of topical formulations containing OMC and BMBM loaded into lipid-based nanoparticles.

In conclusion, both the vehicle composition and the pattern of application affected BMBM and OMC skin permeation. However, all formulations investigated in this study could be considered safe under in-use conditions.

## Figures and Tables

**Table 1:** Composition (% *w*/*w*) of oil in water (O/W) emulsions 1 and 2. IPM: isopropyl myristate; OMC: octylmethoxycinnamate; BMBM: butylmethoxydibenzoylmethane; GMS: glyceryl monostearate.

Ingredient	1	2
Phase A		
Myritol 318	1.60	1.60
IPM	2.40	2.40
Cetiol V	3.00	3.00
Cetiol SN	5.00	5.00
Beeswax	1.00	0.70
OMC	5.00	5.00
BMBM	1.00	1.00
Emulgade SE	5.50	5.00
GMS	0.50	0.35
Phase B		
Disodium EDTA	0.10	0.10
Glycerin	5.00	5.00
Water	q.s.100	q.s.100
Phase C		
Gram 1	0.35	0.35
Kathon CG	0.05	0.05

**Table 2:** Composition (% *w*/*w*) of water in oil (W/O) emulsion 3.

Ingredient	% *w*/*w*
Phase A	
Mineral oil	13.0
Squalane	3.00
GMS	1.00
Sorbitan monostearate	7.00
BHT	0.05
OMC	5.00
BMBM	1.00
Phase B	
Disodium EDTA	0.10
Water	q.b.100
Glycerin	5.00
Phase C	
Gram 1	0.35
Kathon CG	0.05

**Table 3:** Composition (% *w*/*w*) of oil vehicles 4–6 and required hydrophilic lipohilic balance (HLB_r_) valuesof oil ingredients. All vehicles contained 1.0% *w*/*w* BMBM, 5.0% *w*/*w* OMC and 0.05% *w*/*w* BHT.

Oil	HLB_r_	Vehicle		
		4	5	6
Mineral oil	4, 5	83.95	69.95	49.95
Almond oil	6	5.00	5.00	5.00
Jojoba oil	6	5.00	5.00	5.00
IPM	11	0.00	14.00	34.00

**Table 4:** In vitro release of OMC and BMBM from vehicle 1–6: cumulative amount released after 24 h (Q_24_ ± S.D.), percentage of dose released after 24 h (% D), release rate (flux ± S.D.) and lag time.

Vehicle	UV-Filter	Q_24_ ± S.D. (μg/cm^2^)	% Dose	Flux (μg/cm^2^/h)	Lag Time (h)
1	OMC	73.41 ± 6.47	7.34	3.09 ± 0.28	0.20
	BMBM	1.36 ± 0.11	0.68	N.DT.	N.DT.
2	OMC	75.83 ± 7.77	7.58	3.20 ±0.29	0.11
	BMBM	1.29 ±0.09	0.65	N.DT.	N.DT.
3	OMC	90.66 ± 8.31	9.07	3.80 ± 0.35	0.15
	BMBM	1.46 ± 0.15	0.73	N.DT.	N.DT.
4	OMC	72.56 ± 8.02	7.26	3.07 ± 0.35	0.41
	BMBM	N.D. ^1^	N.DT. ^2^	N.DT.	N.DT.
5	OMC	95.23 ± 8.99	9.52	4.02 ± 0.39	0.17
	BMBM	1.03 ± 0.10	0.51	N.DT.	N.DT.
6	OMC	1.27 ± 0.14	0.13	N.DT.	N.DT.
	BMBM	N.D.	N.DT.	N.DT.	N.DT.

^1^ N.D. = Not detectable in the receptor phase; ^2^ N.DT. = not determined because no UV-filter was detected in the receptor phase up to 8 h.

**Table 5:** OMC and BMBM physicochemical properties: molecular weight (MW), topological polar surface area (TPSA), melting point (MP), water solubility and log partition coefficient octanol/water (Log K_ow_). Data obtained from Pubchem, 2017 [46].

Parameter	OMC	BMBM
MW (g/mol)	290.403	310.393
TPSA (A^2^)	35.5	43.4
MP (°C)	−68.3	83.5
Water solubility (mg/L)	0.22–0.75 (at 21 °C)	2.2 (at 25 °C)
Log K_ow_	6.10	4.51

**Table 6:** In vitro skin permeation of OMC and BMBM from vehicle 1–6 after a single application: cumulative amount permeated after 24 h (Q_24_ ± S.D.), percentage of dose permeated after 24 h (% D), flux through the skin (flux ± S.D.) and lag time.

Vehicle	UV-Filter	Q_24_ ± S.D. (μg/cm^2^)	% Dose	Flux (μg/cm^2^/h)	Lag Time (h)
1	OMC	27.24 ± 3.23	2.72	3.09 ± 0.28	0.20
	BMBM	1.04 ± 0.10	0.52	N.DT.^2^	N.DT.
2	OMC	27.50 ± 3.96	2.75	3.20 ±0.29	0.11
	BMBM	1.06 ±0.09	0.53	N.DT.	N.DT.
3	OMC	29.85 ± 4.14	2.99	3.80 ± 0.35	0.15
	BMBM	0.57 ± 0.15	0.28	N.DT.	N.DT.
4	OMC	16.64 ± 2.37	1.66	3.07 ± 0.35	0.41
	BMBM	N.D. ^2^	N.DT.	N.DT.	N.DT.
5	OMC	46.63 ± 6.91	4.66	4.02 ± 0.39	0.17
	BMBM	0.73 ± 0.21	0.36	N.DT.	N.DT.
6	OMC	N.D. ^1^	N.DT.	N.DT.	N.DT.
	BMBM	N.D.	N.DT.	N.DT.	N.DT.

^1^ N.D. = Not detectable in the receptor phase; ^2^ N.DT. = not determinable.

**Table 7:** In vitro skin permeation of OMC and BMBM from vehicle 1–6 after repeated applications for three consecutive days: cumulative amount permeated after 24 h (Q_24_ ± S.D.) and percentage of dose permeated after 24 h (% D).

Vehicle	UV-Filter	Q_24_ ± S.D. (µg/cm^2^)			% Dose		
		Day 1	Day 2	Day 3	Day 1	Day 2	Day 3
1	OMC	13.45 ± 1.98	16.78 ± 2.03	16.23 ± 3.07	1.35	1.57	1.62
	BMBM	0.56 ± 0.03	0.70 ± 0.02	0.72 ± 0.10	0.28	0.35	0.36
2	OMC	13.03 ± 2.31	17.33 ± 2.97	17.46 ± 4.33	1.30	1.73	1.74
	BMBM	0.55 ± 0.02	0.72 ± 0.23	0.71 ±0.20	0.27	0.36	0.36
3	OMC	13.99 ± 1.76	15.00 ± 2.49	15.41 ± 2.98	1.40	1.50	1.54
	BMBM	N.D. ^1^	N.D.	N.D.	N.DT. ^2^	N.DT.	N.DT.
4	OMC	8.09 ± 1.02	9.55 ± 2.10	8.93 ± 1.29	0.81	0.95	0.89
	BMBM	N.D.	N.D.	N.D.	N.DT.	N.DT.	N.DT.
5	OMC	26.44 ± 3.71	29.36 ± 5.99	28.14 ± 4.15	2.64	2.93	2.81
	BMBM	N.D.	N.D.	N.D.	N.DT.	N.DT.	N.DT.
6	OMC	N.D.	N.D.	N.D.	N.DT.	N.DT.	N.DT.
	BMBM	N.D.	N.D.	N.D.	N.DT.	N.DT.	N.DT.

^1^ N.D. = Not detectable in the receptor phase; ^2^ N.DT. = not determinable.

**Table 8:** Amount (μg/cm^2^) of OMC delivered from the skin in the receiving medium after the removal of the vehicle in the repeated application protocol. N.D. = Not detectable in the receptor phase.

Vehicle	Day 1	Day 2	Day 3
1	4.5 ± 0.5	7.7 ± 1.5	7.3 ± 1.6
2	3.9 ± 0.3	8.3 ± 2.0	8.4 ± 1.9
3	4.8 ± 0.4	5.8 ± 1.0	6.2 ± 1.7
4	3.5 ± 0.4	5.0 ± 0.9	4.4 ± 1.0
5	11.5 ± 1.8	14.5 ± 2.1	13.3 ± 1.9
6	N.D.	N.D.	N.D.

**Table 9:** SED and MoS values of OMC and BMBM determined after a single application (single) or repeated applications (repeated) for three consecutive days of vehicles 1–6. N.D. = Not determinable.

Vehicle	UV-Filter	SED (mg/Kg/day)				MoS			
		Single	Repeated			Single	Repeated		
			Day 1	Day 2	Day 3		Day 1	Day 2	Day 3
1	OMC	6.81	3.36	4.17	4.05	66	134	108	111
	BMBM	0.26	0.14	0.17	0.18	1730	3214	2647	2500
2	OMC	6.86	3.25	4.33	4.36	66	138	104	103
	BMBM	0.27	0.14	0.18	0.18	1666	3214	2500	2500
3	OMC	7.46	3.50	3.75	3.85	60	129	120	117
	BMBM	0.14	N.D.	N.D.	N.D.	3214	N.D.	N.D.	N.D.
4	OMC	4.16	2.02	2.39	2.23	108	223	188	202
	BMBM	N.D.	N.D.	N.D.	N.D.	N.D.	N.D.	N.D.	N.D.
5	OMC	11.66	6.61	7.34	7.03	39	68	61	64
	BMBM	0.18	N.D.	N.D.	N.D.	2500	N.D.	N.D.	N.D.
6	OMC	N.D.	N.D.	N.D.	N.D.	N.D.	N.D.	N.D.	N.D.
	BMBM	N.D.	N.D.	N.D.	N.D.	N.D.	N.D.	N.D.	N.D.
